# Weight loss in children undergoing allogeneic hematopoietic stem cell transplantation within the first 100 days: Its influencing factors and impact on clinical outcomes

**DOI:** 10.3389/fnut.2022.974389

**Published:** 2023-01-09

**Authors:** Mei Yan, Jian Pan, Jie Huang, Changwei Liu, Xiaona Xia, Ting Zhu, Yuanyuan Wan, Yongjun Fang, Weibing Tang

**Affiliations:** ^1^Department of Clinical Nutrition, Children’s Hospital of Nanjing Medical University, Nanjing, China; ^2^Department of Hematology and Oncology, Children’s Hospital of Nanjing Medical University, Nanjing, China

**Keywords:** pediatric, weight (mass), allo and autologous transplantation, screening tools, graft versus host disease

## Abstract

**Purpose/Objective:**

This study aimed to evaluate the nutritional status of children subjected to allogeneic hematopoietic stem cell transplantation (alloHSCT) in the first 100 days. Objectives were to clarify the effect of weight loss on clinical outcomes, and to analyze factors influencing weight loss.

**Methods:**

Eighty pediatric patients receiving alloHSCT were enrolled in the study. Body mass index (BMI) z-scores and weight for age (WFA) z-scores were collected. A multivariate regression model was set up to investigate factors affecting weight loss. Post-transplant clinical outcomes relative to weight loss on 100 days after transplantation were analyzed.

**Results:**

At admission, eight patients (10%) were underweight, the number had increased to 23 (30.67%) by 100 days post-HSCT. On day + 100, only nutrition screening tool for childhood cancer (SCAN) scores ≥ 3 (OR: 4.474, 95% CI: 1.215, 16.472; *P* = 0.024) and acute graft versus host disease (aGVHD) (OR: 9.915, 95% CI: 3.302, 29.771; *P* < 0.001) were regarded as significant influencing factors of weight loss. The Weight loss ≥ 5% group was associated with longer hospital stays (*P* = 0.001), greater cost of inpatient treatment (*P* = 0.001), and a higher incidence of 100-day re-admission and intensive care unit (ICU) transfer (*P* = 0.03 and *P* = 0.033, respectively). Cumulative number of fever days (*P* = 0.023) and antibiotic use (*P* = 0.007) also increased significantly. The Weight loss ≥ 5% group had a significantly lower one-year overall survival rate compared with the Weight loss < 5% group (*P* = 0.015).

**Conclusion:**

Pediatric patients’ nutritional status declined significantly after HSCT. Weight loss within the first 100 days influenced short-term clinical outcomes and one-year overall survival.

## Introduction

Allogeneic hematopoietic stem cell transplantation (alloHSCT) is a complex and effective therapy for hematologic malignant and non-malignant diseases in adult and pediatric patients (1). However, myeloablative (MAC) or reduced intensity conditioning (RIC) regimens and complications of alloHSCT often cause patients undergoing HSCT to experience loss of appetite, mucositis, vomiting, and diarrhea (2). Acute graft versus host disease (aGVHD) is a major complication of alloHSCT. It is an immune disorder characterized by disrupting particular organs, consisting of the skin, gastrointestinal tract and liver, which can lead to insufficient caloric intake (3). Energy and protein requirements increase in HSCT because of the intense catabolism and the demands of physical growth in children (4). Thus, malnutrition and weight loss are common in pediatric patients.

Malnutrition is usually assessed by body mass index (BMI) and body weight for age (WFA) z-scores. BMI and weight typically decline consistently following HSCT (5). Weight loss were reported in patients at different periods of alloHSCT. A previous study showed almost 70% of adult patients had weight loss greater than 5% within 30 days (6). Urbain et al. reported that 23.8% adult patients at admission had significant weight loss (>5%) in the previous 6 months (7). Fuji found 63.5% (92/145) patients lost weight ≥ 5% during HSCT (8). Nutritional status is constantly changing. However, in clinical practice, many physicians are not aware of the subtle changes that contribute to weight loss over time.

Some studies have shown that weight loss in pediatric patients undergoing HSCT is associated with poor outcomes (9), and these works mainly focused on weight loss before HSCT. Weight loss following transplantation has been studied less in children. And questions remain about whether weight loss post-transplantation is associated with clinical outcomes, especially in children. Thus, the present study had two aims. First, we set out to document the nutritional status of pediatric patients with alloHSCT. Second, we aimed to evaluate the impact of weight loss on the clinical outcomes after alloHSCT, and to analyze factors affecting weight loss after HSCT. Ultimately, we sought to contribute information on appropriate timing and nutritional support to improve clinical outcomes for pediatric patients undergoing alloHSCT.

## Materials and methods

### Participants

Children were involved in this study if they were younger than 18 years, and had undergone alloHSCT at Children’s Hospital of Nanjing Medical University between April 2018 and April 2022.

To prevent the occurrence of aGVHD, all pediatric patients received intravenous cyclosporine (CsA) at a dose of 1∼3 mg/kg/day combined with methotrexate (MTX), 15 mg/m^2^ given on day 1 and 10 mg/m^2^ on day 3 and 6. When grade II to IV aGVHD presents, methylprednisolone (2 mg/kg/day) along with CsA was added. If glucocorticoid resistance occurred, tacrolimus, antithymocyte globulin, mycophenolate mofetil and other second-line drugs should be added.

Dieticians regularly evaluated the dietary intake of children undergoing allo-HSCT. Children received EN and/or PN if their dietary intakes were insufficient.

Patients were grouped into a Weight loss < 5% group and a Weight loss ≥ 5% group. Weight loss after alloHSCT was operationally defined as the relative difference (%) between weight at engraftment and minimum weight over day 100 post-HSCT, or one week before death if patients died within the 100-day period.

The study was approved by the Ethics Committee of the Children’s Hospital of Nanjing Medical University.

### Assessment of nutritional status

Body weight and height were collected at admission and on the day of transplantation, and then on days 7 (day + 7), 14 (day + 14), 21 (day + 21), 30 (day + 30), and 100 (day + 100) days post-HSCT. BMI and WFA z-scores of patients under 5-years of age were calculated using WHO Anthro, and WHO AnthroPLUS for children older than 5.^1^ Nutritional status was categorized into three groups as follows: underweight (BMI z-scores ≤ −2), average-weight (−2 < BMI z-scores < 2), overweight or obese (BMI z-scores ≥ 2).

### Nutrition screening tool for childhood cancer (SCAN)

Screening tool for childhood cancer is a nutritional screening tool focusing on children with cancer (10). It is based on six questions: high risk cancer, intensive treatment, gastrointestinal symptoms, poor oral intake, weight loss, and signs of under nutrition. Score of 1 or 2 is for each question, with a maximum score of 10 points. Scores ≥ 3 indicate children are at risk of malnutrition and need dieticians for further evaluation.

### Definitions of transplantation parameters

Acute graft versus host disease (aGVHD) was graded according to symptoms of skin, liver, and the gut, as documented in previous work (11). Clinical outcomes included the duration of hospital stay, and the costs of inpatient treatment during hospitalization. The duration of antibiotic and corticosteroid use, cumulative number of days with fever were calculated from transplantation to hospital discharge. Other outcomes were transfer to the intensive care unit (ICU) during hospitalization and hospital re-admissions within 100 days post-transplantation. Overall survival (OS) was defined as time from engraftment to death from any reason within one year after alloHSCT. Treatment-related mortality (TRM) was defined as death without relapse or disease progression within one year after alloHSCT. Cumulative incidence of relapse (CIR) was defined as time to recurrence of disease within one year after alloHSCT.

### Statistical analysis

SPSS 17.0 software and Stata 16 were used for data analysis. We used the median (P25, P75) [M (P25, P75)] to describe data that was not normally distributed, and mean ± standard deviation to describe normally-distributed data. Repeated measurements analysis of Variance (RM-ANOVA) was used for multigroup comparison. The Mann–Whitney U test was used to compare two groups with non-normal data distribution. The Chi square test was used to compare categorical data. To identify the influence of potential factors on weight loss, we used binary logistic regression models with weight loss (yes or no) as the dependent variable. First, univariate models were used to assess single variable. Then, a multivariate model was built containing three variables (donor status, aGVHD, SCAN). The probability and curve of OS and TRM were computed using Kaplan–Meier survival analysis. A Cox proportional hazards regression model was applied to analyze OS. Univariate model was used to assess single variable. Multivariate model was built by choosing covariates with *P* < 0.1 in the univariate model. Directed acyclic graph is used for the chosen of confonders in multivariable analysis. We used the method of Fine and Gray for univariate and multivariate analysis of TRM and CIR. In the competing risk models for TRM, CIR was defined as a competing risk. Patients who could not be followed up were tracked in terms of their final follow-up data and were analysed as censored data. *P* < 0.05 was considered as statistically significant.

## Results

### Patient characteristics

Characteristics of the children undergoing alloHSCT are exhibited in Table 1. Eighty children undergoing alloHSCT were included in the study. The sample comprised 61.25% male and 38.75% female patients, and the median age was 5.75 years. The most common donor type was matched unrelated donors (56.25%). Fifty-seven patients (71.25%) received the MAC regimen while 23 (28.75%) followed the RIC regimen. At admission, 69 patients (86.25%) had a BMI within the normal range, while 10% were underweight, and 3.75% were overweight or obese. At engraftment, 66 patients (82.5%) were of an average weight while 13.75% were underweight, and 3.75% were overweight or obese. The number of underweight patients rose to 23 (30.67%) at day + 100 post-HSCT. The mean weight loss was 0.55 ± 2.51 kg, and 15 patients (18.75%) lost 5–10% body weight with 20 (25%) losing over 10% weight. The primary diagnoses were aplastic anemia (25%) and acute myeloid leukemia (23.75%). Other diagnoses included acute lymphoid leukemia, myelodysplastic syndrome, juvenile granular monocytic leukemia, acute biphenotypic leukemia, congenital thrombocytopenia, immunodeficiency disease, thalassemia, dyskeratosis congenita, and so on. 62.5% (50/80) of the patients were diagnosed with aGVHD following HSCT, Grade II-IV aGVHD occurred in 33 patients (41.25%). Cumulative incidences of aGVHD in each organ: skin 17 (21.25%), gut 31 (38.75%), liver 2 (2.5%). At admission, 20 patients (25%) were with SCAN scores ≥ 3.

**TABLE 1:** Characteristics of child participants in the study.

Characteristic		
Median age, years (P25–P75)		5.75 (2.77–9.61)
**Sex, *n* (%)**		
	Female	31 (38.75)
	Male	49 (61.25)
**Diagnosis, *n* (%)**	**Malignant diseases**	
	Acute myeloid leukemia	19 (23.75)
	Acute lymphoid leukemia	4 (5.00)
	Juvenile granular monocytic leukemia	2 (2.50)
	Myelodysplastic syndrome	2 (2.50)
	Acute biphenotypic leukemia	2 (2.50)
	Chronic granulocytic leukemia	3 (3.75)
	Hemophagocytic syndrome	4 (5.00)
	EBV associated T-cell leukemia	1 (1.25)
	Neuroblastoma	1 (1.25)
	**Non-malignant diseases**	
	Aplastic anemia	20 (25.00)
	Immunodeficiency disease	8 (10.00)
	Thalassemia	3 (3.75)
	Dyskeratosis congenita	1 (1.25)
	Mucopolysaccharidosis	4 (5.00)
	Chronic granulomatous disease	1 (1.25)
	Adrenoleukodystrophy	1 (1.25)
	Congenital thrombocytopenia	2 (2.50)
	Congenital neutropenia	1 (1.25)
	Fucosidosis	1 (1.25)
**Type of transplant, *n* (%)**		
	Matched Related	35 (43.75)
	Matched Unrelated	45 (56.25)
**Conditioning regimens, *n* (%)**		
	MAC	57 (71.25)
	RIC	23 (28.75)
**BMI at admission, *n* (%)**		
	Underweight	8 (10.00)
	Average weight	69 (86.25)
	Overweight or obese	3 (3.75)
**BMI at engraftment, *n* (%)**		
	Underweight	11 (13.75)
	Average weight	66 (82.5)
	Overweight or obese	3 (3.75)
**BMI at day + 100 post-HSCT, *n* (%)**		
	Underweight	23 (30.67)
	Average weight	50 (66.67)
	Overweight or obese	2 (2.66)
**SCAN at admission, *n* (%)**		
	Score ≥ 3 points	20 (25.00)
	Score < 3 points	60 (75.00)
**Weight loss after 100 days post-HSCT, *n* (%)**		
	Mean ± SD (kg)	0.55 ± 2.51
	<5% weight loss	45 (56.25)
	5–10% weight loss	15 (18.75)
	>10% weight loss	20 (25.0)
**aGVHD, *n* (%)**		
	Grade 0-I aGVHD	47 (58.75)
	Grade II-IV aGVHD	33 (41.25)
acute gastrointestinal GVHD		31 (38.75)
acute Hepatic GVHD		17 (21.25)
acute cutaneous GVHD		2 (2.5)

MAC, myeloablative regimens; RIC, reduced intensity conditioning regimens; BMI, body mass index; HSCT, hematopoietic stem cell transplantation; SCAN, nutrition screening tool for childhood cancer; Grade II-IV aGVHD, moderate and severe acute graft versus host disease; and Grade 0-I aGVHD, none and mild acute graft versus host disease.

### Nutritional assessment of children before and after alloHSCT

**FIGURE 1:**
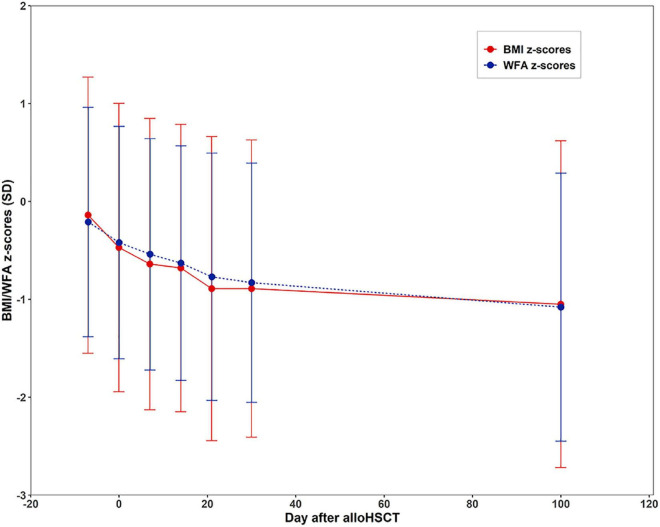
Changes of mean body mass index (BMI) z-scores and weight for age (WFA) z-scores during treatment of alloHSCT (at initial admission and the day of transplantation, day + 7, day + 14, day + 21, day + 30, and day + 100 after HSCT).

Patients’ BMI and WFA z-scores are shown in Figure 1. The longitudinal data reveals that both BMI z-scores (F = 16.467, *P* < 0.001) and WFA z-scores (F = 21.07, *P* < 0.001). BMI z-scores declined from −0.14 ± 1.41 at admission to −0.47 ± 1.47 at engraftment, and the minimum level observed was −1.05 ± 1.67 at day + 100. Simultaneously, WFA z-scores declined from −0.22 ± 1.17 at admission to −0.42 ± 1.19 at engraftment, and the minimum level observed was −1.08 ± 1.37 on day + 100.

### Impact of factors influencing weight loss

Univariate analysis indicated that donor status (*P* = 0.018), aGVHD (*P* < 0.001), oral mucositis (*P* = 0.014), duration of corticosteroid (*P* = 0.001) and Nutrition screening tool for childhood cancer (SCAN) scores ≥ 3 (*P* = 0.002) were strongly associated with weight loss ≥ 5%. A multivariate regression model was developed with variables (donor status, aGVHD, SCAN) displaying a connection (*P* < 0.1) with weight loss. However, only SCAN scores ≥ 3 (OR: 4.474, 95% CI: 1.215, 16.472; *P* = 0.024) and presence of ≥grade II acute GVHD (OR: 9.915, 95% CI: 3.302, 29.771; *P* < 0.001) were recognized as independent effect factors of weight loss in the 100 day period following HSCT (Table 2).

**TABLE 2:** Results of univariate and multivariate analyses of factors affecting weight loss ≥ 5%.

Univariate analysis			
Variables		OR (95% CI)	*P*-value
Age at admission	<5 years old vs. ≥5 years old	1.125 (0.459–2.758)	0.797
Sex	Female vs. Male	1.684 (0.679–4.180)	0.261
Diagnosis	Non-malignant diseases vs. Malignant diseases	0.615 (0.253–1.499)	0.285
Donor status	Unrelated vs. Related	3.125 (1.221–8.00)	0.018
Conditioning chemotherapy	Reduced intensity vs. Myeloablative	0.766 (0.286–2.055)	0.597
BMI at admission	Underweight vs. Average weight or Overweight	0.394 (0.074–2.085)	0.273
Oral mucositis	Positive vs. Negative	3.273 (1.266–8.458)	0.014
SCAN at admission	Scores ≥ 3 vs. Scores < 3	6.000 (1.908–18.867)	0.002
aGVHD	II-IV vs. 0-I	11.562 (4.009–33.345)	<0.001
Duration of corticosteroid		1.032 (1.012–1.052)	0.001
**Multivariate analysis**			
SCAN at admission	Scores ≥ 3 vs. Scores < 3	4.474 (1.215–16.472)	0.024
aGVHD	II-IV vs. 0-I	9.915 (3.302–29.771)	<0.001

Duration of corticosteroid is linear categories. SCAN, nutrition screening tool for childhood cancer; Grade II-IV aGVHD, moderate and severe acute graft versus host disease; and Grade 0-I aGVHD, none and mild acute graft versus host disease.

### Post-transplant clinical outcomes relative to weight loss 100 days after transplantation

The length of hospital stay was longer in the Weight loss ≥ 5% group than the Weight loss < 5% group (*P* = 0.001) (Median: 83.0 vs. 65.0 days; Quartile: 67–110 vs. 55.5–79.5). Patients in the Weight loss ≥ 5% group had higher costs of inpatient treatment (Median: 239254.72 vs. 198188.38 CNY; Quartile: 201517.55–345488.63 vs. 160378.865–234428.355) than the Weight loss < 5% group. A longer duration of cumulative febrile episodes (*P* = 0.023), and antibiotic use (*P* = 0.007) were observed in the Weight loss ≥ 5% group. A higher incidence of 100-day re-admission (*P* = 0.03) and ICU transfer (*P* = 0.033) was also noted (Table 3).

**TABLE 3:** Post-transplant clinical outcomes relative to weight loss after transplantation.

Variable (*n* = 80)	Weight loss ≥ 5% (*n* = 35)	Weight loss < 5% (*n* = 45)	Parameter estimate	*P*-value
Median duration of hospital stay (IQR), Days	83 (67, 110)	65 (55.5, 79.5)	Z = −3.245	0.001
Median costs of inpatient treatment (IQR), CNY	239254.72 (201517.55,345488.63)	198188.38 (160378.865,234428.355)	Z = −3.302	0.001
Median length of cumulative febrile episodes (IQR), Days	10 (5, 19)	7 (2, 13)	Z = −2.279	0.023
Median duration of Antibiotic Use (IQR), Days	42 (30.0, 72.0)	33 (20.0, 48.5)	Z = −2.712	0.007
100-day re-admission, *n* (%)	21 (60)	16 (35.6)	χ^2^ = 4.732	0.030
ICU transfer, *n* (%)	8 (22.86)	2 (4.44)	χ^2^ = 4.535	0.033

ICU, intensive care unit; CNY, Chinese Yuan; and IQR, interquartile range.

**FIGURE 2:**
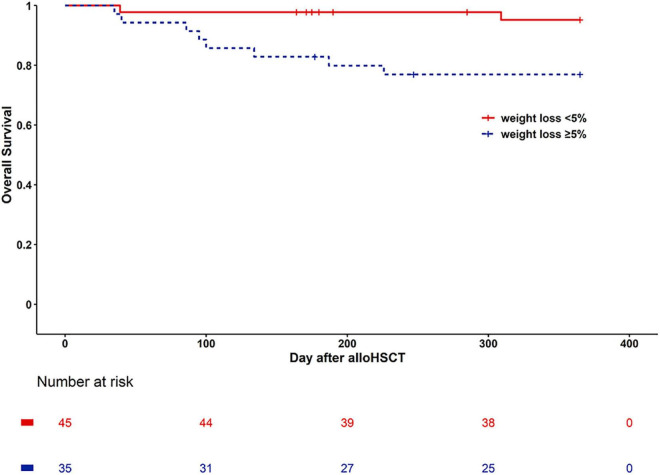
Kaplan Meier curves of survival for weight loss < 5% group (continuous line) and weight loss ≥ 5% (dashed line).

There are 10 patients died in one year after alloHSCT. Two patients died of refractory disease progression or relapses. 8 of 10 patients died of transplant-associated complications, the causes of death were pulmonary infection (*n* = 4), heart failure (*n* = 1), aGVHD-related (*n* = 3). Kaplan Meier curves of one-year overall survival for the Weight loss < 5% group and the Weight loss ≥ 5% group indicated survival of 95.6 versus 77.1%, and means of 355.93 versus 306.72 days, respectively (Log rank, *P* = 0.015) (Figure 2). The Cox multivariate analysis revealed that Weight loss ≥ 5% (HR: 5.585, 95% CI: 1.183, 26.357; *P* = 0.03) and relapse (HR: 6.315, 95% CI: 1.285, 31.039; *P* = 0.023) had significant impacts on OS (Supplementary Table 1). One year OS for Weight loss < 5% group and Weight loss ≥ 5% groups was 97.4 versus 84.6%, respectively (*P* = 0.045) after excluding patients who died or suffered from relapse/progression within 100 days (Supplementary Figure 1).

**FIGURE 3:**
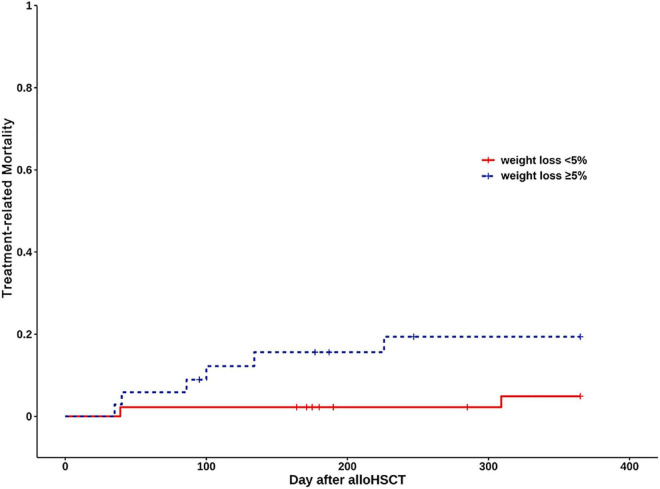
Kaplan Meier curves of treatment-related mortality (TRM) for weight loss < 5% group (continuous line) and weight loss ≥ 5% (dashed line).

The cumulative incidence of TRM at one year was 17.1% in the Weight loss ≥ 5% group, 4.4% in the Weight loss < 5% group (Log rank, *P* = 0.058) (Figure 3). Weight loss ≥ 5% was not associated with increased TRM (HR: 4.07, 95% CI: 0.83, 20.03; *P* = 0.084). After adjustment for age, diagnosis, gender, donor type, conditioning regimen, and aGVHD, weight loss ≥ 5% was not associated with an increased risk of TRM (HR: 3.65, 95% CI: 0.60–22.12; *P* = 0.159) (Supplementary Figure 2).

## Discussion

In this study, 10% of patients were underweight in the pre-HSCT stage. These findings align with previous literature where most children are well-nourished, and the prevalence of underweight children ranges from 4 to 16.3% (12, 13). In previous studies, the percentage of overweight children ranges from 12 to 15% (12, 14). However, our study showed only three children (3.75%) were overweight or obese. This indicates that children undergoing HSCT in China might have a lower rate of over nutrition than their peers in developed countries. Inaba et al. (15) concluded that BMI z-scores in children with hematologic malignancies after alloHSCT declined significantly over time. Similarly, Campos et al. (5) identified a significant BMI decrease in pediatric patients in the 6-month period post-HSCT. We found that nutritional status of children deteriorated gradually in the 100 days after HSCT, with the lowest BMI z-scores on day 100. Weight loss (≥5%) was observed in 35 patients (43.75%) in our sample.

Nutritional status was traditionally assessed by anthropometric and laboratory measurements. In recent years, body composition measurement has become an important indicator reflecting nutritional status of pediatric patients (16). However, in developing countries, its application is restricted as the measuring instrument includes bioelectrical impedance analysis (BIA) and Dual-energy X-ray absorptiometry (DXA)–tools which are expensive and often impractical in such settings. Albumin levels can also be affected by several non-nutritional factors (15). Anthropometry mainly includes BMI and weight. Children are split into four groups: normal, wasted, severely wasted, overweight or obesity on the basis of BMI/WFA z-scores. BMI levels at different stages of HSCT have different effects on survival. However, it only represents weight in relation to height, BMI z-scores remain unchanged in the course of treatment, possibly because weight and height z-scores both decrease. Therefore, BMI/WFA z-scores alone are not very reliable to evaluate nutritional status. It should combine with weight and height change to monitor nutritional status. In comparison, periodic measurements of body weight are an easy and convenient way to assess nutritional status and can effectively contribute to nutritional improvement.

We analyzed factors that might influence weight loss in the 100-day period in children undergoing alloHSCT. Oral mucositis and ≥grade II aGVHD were found to have a significant effect on weight loss over day 100. Oral mucositis is a common complication of alloHSCT, resulting from the conditioning regimen and radiotherapy. It leads to destruction of the oral mucosal barrier and impairs oral intake because of chewing and swallowing difficulties (16). Fat-free mass index, and muscle mass demonstrate a significant decline in adult patients with severe mucositis (17). Feng et al. (18) found that glucocorticoid treatment and mucositis occurrence affected BMI z-scores and arm muscle area index in children undergoing allogeneic HSCT. Eduardo et al. (19) showed that oral mucositis was not an independent factor for weight loss in adult patients undergoing alloHSCT but was an independent factor for weight gain in HSCT. In our study, oral mucositis was strongly correlated with weight loss ≥ 5% in the univariate analysis, but was not pinpointed as an independent factor in the multivariate regression. This finding may have been restricted by the limited number of patients in our sample. Aggressive treatment of mucositis in patients starting to develop the condition may help improve their nutritional intake and prevent deterioration in nutritional status.

Urbain et al. (7) found that presence of ≥grade II aGVHD was an independent factor influencing weight loss in adults. Similarly, El-Ghammaz et al. (20) showed that nutritional status of adult patients experiencing ≥grade II aGVHD worsened during hospitalization and after discharge. Our study also supports these findings–presence of ≥grade II aGVHD had a significant influence on weight loss by day 100, according to the multivariate analysis. Corticosteroid and immunosuppressive drugs used for treatment of aGVHD have negative effects on muscle metabolism after HSCT (21). Some studies indicated that fat-free tissues in children with aGVHD after alloHSCT were more likely to be affected (18). Most children with aGVHD are not physically active, compounding the muscle degradation from steroids. Previous research demonstrated a significant correlation between physical function and quality of life in patients receiving HSCT (22). Therefore, prevention and treatment of aGVHD must be prioritized because of its high morbidity. Nutritional and exercise interventions are an important component of treatment for children with aGVHD.

According to ASPEN, all patients undergoing HSCT are at nutrition risk and should screening nutritional risk (23). There are some nutrition screening tools focus on children, but none are able to reach all the requirements of a cancer special tool. SCAN according to Murphy et al. (10) offers a simple way to identify pediatric patients with cancer who are already malnourished or at risk of malnutrition. It considers cancer type and treatment, not only nutrition related symptoms. To date, there are few studies of the application of SCAN. In our study, SCAN was shown to have prognostic associations with weight loss ≥ 5% after transplantation. SCAN is, thus, recommended as a key part of the comprehensive assessment of nutritional status in children undergoing HSCT. Children with SCAN scores ≥ 3 are at risk of malnutrition and need periodical assessment of nutrition.

Weight loss is included in some nutritional screening tools. For example, STRONG kids (24) includes weight loss as a main aspect, although the degree of weight loss has not been quantified. Fuji et al. (8) found that weight loss in adult patients undergoing HSCT has a significant effect on clinical outcomes, after assessing BMI and weight loss as part of nutritional screening. Ando et al. (9) noted that weight loss in the period from diagnosis to transplant was associated with worse OS and graft-versus-host disease-free survival for adults with acute myeloid leukemia following HSCT. Weight loss > 7% during alloHSCT (from admission to the first outpatient visit) was associated with increased time in hospital (25). These reports focus on weight loss in adult populations, but comparable data for children post-transplantation are scarce. The present study revealed that weight loss in children undergoing HSCT was associated with longer hospital stays, longer duration of antibiotic use. Children in the Weight loss ≥ 5% group also experienced greater duration of cumulative febrile episodes than the Weight loss < 5% group. Weight loss after alloHSCT was connected with lower OS. It also has higher TRM, although there is of no statistical significance. Thus, it is rational to use weight loss ≥ 5% in the 100 days following HSCT to identify pediatric patients at risk of poor clinical outcomes. Moreover, weight loss remain easy and effective ways to recognized patients at nutritional risk.

If inadequate oral intake or weight loss is detected, children should accept nutritional support so as to maintain their nutritional status. The Harris-Benedict formula is usually used to determine the target caloric intake (26). Enteral nutrition (EN) is always favored as the first option compared to parenteral nutrition (PN). PN was administered mainly in cases of gastrointestinal failure. However, in clinical practice, PN is often preferred over EN owing to practical reasons such as the intolerance of nasogastric tubes. In our study, 43 cases received PN, and 39 cases received EN. Moreover, the utilization of EN is usually short-term and discontinuous. Nutritional counsel and education of patients and their parents are important. Children undergoing alloHSCT suffer from weight loss which was associated with poor clinical outcomes. Thus, nutritional intervention is essential. Aggressive therapy of aGVHD may help prevent weight loss and malnutrition. In addition, exercise interventions are an important component of preventing weight loss.

The limitations of our study were a small sample size, which leading to the wide confidence intervals of the variables in binary logistic regression model, and lack of detailed nutritional intervention. Future work should include a larger sample together with effective nutritional support. The body composition measurements and laboratory indexes (namely albumin/pre-albumin) were also lacking, and should therefore be covered in regression models in future research.

## Data availability statement

The raw data supporting the conclusions of this article will be made available by the authors, without undue reservation.

## Author contributions

MY, JP, JH, YF, and WT took part in the research design. MY, CL, XX, TZ, and YW were participated in the collection and maintenance of datas. MY, JP, and WT analyzed the data and interpreted results. MY, JH, and JP wrote the manuscript. YF and WT revised the manuscript. All authors agreed the article to be published.

## Footnotes

1
http://www.who.int/childgrowth/software/en/

## Supplementary material

Supplementary Figure 1 Kaplan Meier curves of survival for weight loss < 5% group (continuous line) and weight loss ≥ 5% (dashed line) after excluding patients who died or suffered from relapse/progression within 100 days.

Supplementary Figure 2 Cumulative incidence of TRM and relapse in weight loss ≥ 5% group and weight loss < 5% group.
