# Supplementary figures and images for: Weight loss in children undergoing allogeneic hematopoietic stem cell transplantation within the first 100 days: Its influencing factors and impact on clinical outcomes
Source: Front Nutr. 2023 Jan 9;9:974389. doi: 10.3389/fnut.2022.974389 (PMC9868921; doi:10.3389/fnut.2022.974389)

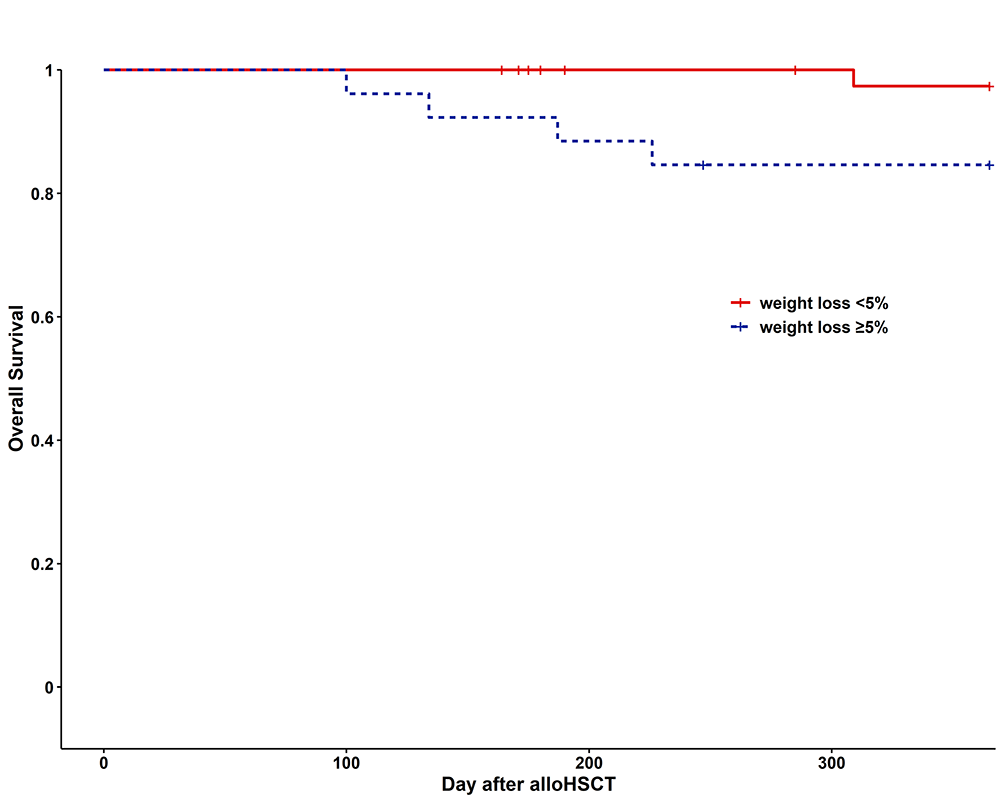

Supplement: Supplementary Figure 1 — Kaplan Meier curves of survival for weight loss < 5% group (continuous line) and weight loss ≥ 5% (dashed line) after excluding patients who died or suffered from relapse/progression within 100 days. [file Image_1.TIF]

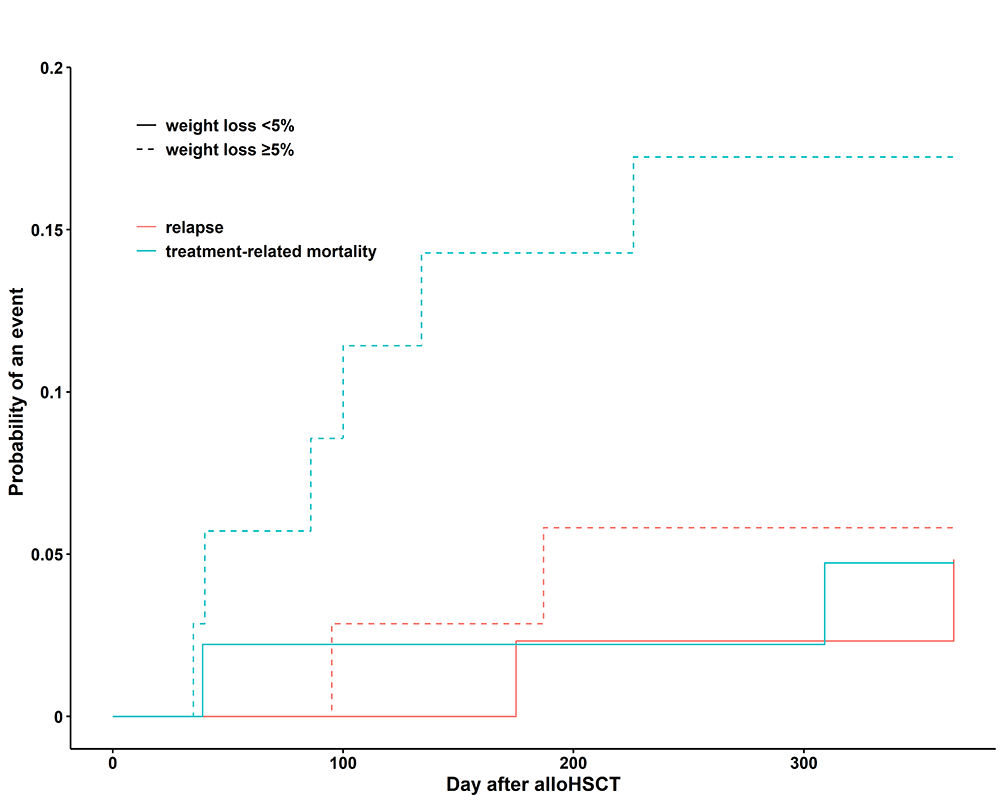

Supplement: Supplementary Figure 2 — Cumulative incidence of TRM and relapse in weight loss ≥ 5% group and weight loss < 5% group. [file Image_2.TIF]
