# Supplementary material for: Weight loss in children undergoing allogeneic hematopoietic stem cell transplantation within the first 100 days: Its influencing factors and impact on clinical outcomes
Source: Front Nutr. 2023 Jan 9;9:974389. doi: 10.3389/fnut.2022.974389 (PMC9868921; doi:10.3389/fnut.2022.974389)
Supplement: Supplementary file 3 [file Table_1.doc]

**Supplemental Table 1**

Results of Univariate and multivariate analyses for overall survival.

| **Univariate analysis** | |  | **Multivariate analysis** | |
| --- | --- | --- | --- | --- |
| **Variable** | **HR (95%CI)** | ***P*-value** | **HR (95%CI)** | ***P*-value** |
| Weight loss ≥5% | 5.553（1.179-26.159） | **0.03** | 5.585（1.183-26.357） | 0.03 |
| Male | 0.593（0.172-2.049） | 0.409 |  |  |
| ≥5 years old | 2.997（0.636-14.115） | 0.165 |  |  |
| Malignant diseases | 2.613（0.675-10.308） | 0.164 |  |  |
| Related | 0.810（0.229-2.871） | 0.744 |  |  |
| MAC | 1.677（0.356-7.898） | 0.513 |  |  |
| Grade II-IV aGVHD | 3.604(0.931-13.941) | **0.063** |  |  |
| Relapse | 6.183(1.288-29.687) | **0.023** | 6.315（1.285-31.039） | 0.023 |

MAC=myeloablative regimens; Grade II-IV aGVHD=moderate and severe acute graft versus host disease.
